# The Danger of Testing by Selecting Controlled Subsets, with Applications to Spoken-Word Recognition

**DOI:** 10.5334/joc.51

**Published:** 2019-01-24

**Authors:** David Liben-Nowell, Julia Strand, Alexa Sharp, Tom Wexler, Kevin Woods

**Affiliations:** 1 Department of Computer Science, Carleton College, Northfield, MN, US; 2 Department of Psychology, Carleton College, Northfield, MN, US; 3 Department of Computer Science, Oberlin College, Oberlin, OH, US; 4 Google, Cambridge, MA, US; 5 Verily Life Sciences, Cambridge, MA, US; 6 Department of Mathematics, Oberlin College, Oberlin, OH, US

**Keywords:** Auditory word processing, Word processing, Statistical analysis, Speech perception, Mathematical modelling

## Abstract

When examining the effects of a continuous variable *x* on an outcome *y*, a researcher might choose to dichotomize on *x*, dividing the population into two sets—low *x* and high *x*—and testing whether these two subpopulations differ with respect to *y*. Dichotomization has long been known to incur a cost in statistical power, but there remain circumstances in which it is appealing: an experimenter might use it to control for confounding covariates through subset selection, by carefully choosing a subpopulation of Low and a corresponding subpopulation of High that are balanced with respect to a list of control variables, and then comparing the subpopulations’ *y* values. This “divide, select, and test” approach is used in many papers throughout the psycholinguistics literature, and elsewhere. Here we show that, despite the apparent innocuousness, these methodological choices can lead to erroneous results, in two ways. First, if the balanced subsets of Low and High are selected in certain ways, it is possible to conclude a relationship between *x* and *y* not present in the full population. Specifically, we show that previously published conclusions drawn from this methodology—about the effect of a particular lexical property on spoken-word recognition—do not in fact appear to hold. Second, if the balanced subsets of Low and High are selected randomly, this methodology frequently fails to show a relationship between *x* and *y* that is present in the full population. Our work uncovers a new facet of an ongoing research effort: to identify and reveal the implicit freedoms of experimental design that can lead to false conclusions.

## Introduction

There is growing concern in psychology and other disciplines that the scientific literature has a much higher rate of false positives than was previously assumed (Ioannidis, 2005; Simmons, Nelson, & Simonsohn, 2011). This fear has grown based on the observation that many published findings fail to replicate (Open Science Collaboration, 2015). The high false-positive rate is attributed, in part, to the tremendous flexibility that researchers have when making methodological and statistical decisions (Asendorpf et al., 2013). For example, researchers make choices throughout the experimental process about whether and how to exclude participants or observations, what covariates to include, how to combine or transform dependent variables, and when to terminate data collection (Wicherts et al., 2016). These “researcher degrees of freedom” provide enough flexibility that, when used opportunistically, even impossible outcomes may be rendered statistically significant (Simmons et al., 2011; Simmons, Nelson, & Simonsohn, 2017).

In some subfields of psychology, experiment design includes deciding which stimuli to present to participants. Given that data collection requires time and other resources, and participants may become frustrated or withdraw from the experiment if testing is excessive, experimenters must make choices about which subset of survey questions, trial types, or stimulus items to include from a larger pool of possible items. Sometimes the choices of which items to include are dictated by prior work (e.g., a shortened form of a personality test that has been validated against a longer form; Donnellan, Oswald, Baird, and Lucas 2006), but often a small subset of items may be selected with the implicit expectation that they represent the population from which they are drawn.

An assumption common to psychological research is that the findings of a particular study should generalize beyond the participants sampled. Concerns about this assumption have gained traction in the literature (Henrich, Heine, & Norenzayan, 2010), and, more recently, there has been a push for researchers to explicitly state and justify the target population for the findings, thus defining the “constraints on generality” (Simons, Shoda, & Lindsay, 2017). Although many researchers, if pressed, might agree that typical research participants (often college students) do not represent the general population, much less attention has been paid to whether the subsets of stimuli selected are representative of the broader population of stimuli from which they have been chosen.

### Approaches to subset selection

When selecting multiple subsets of stimuli to assign to different experimental conditions, researchers often need to control for other relevant variables. For example, to carry out a study on gender stereotyping, Hettinger, Hutchinson, and Bosson (2014) needed to identify two sets of household chores from a longer list—one set to assign to a male character in a story, one to a female character—so that the chosen sets matched on genderedness, pleasantness, difficulty, and time consumption. This approach is used widely in studies of word recognition, the focus of this paper, and has also been used in a variety of other psychological research, including the relationship between race and face perception (Zebrowitz, White, & Wieneke, 2008), between attentional processing and obesity (Carters, Rieger, & Bell, 2015), and between emotion and memory (Schmidt, Patnaik, & Kensinger, 2011), among others. Outside of psychology, this stimulus-selection approach has been used in applications ranging from echocardiographic interpretation (Varga et al., 1999) to deforestation (Jayachandran et al., 2017).

Until recently, selecting matched subsets of items was typically achieved via manual selection by the experimenters themselves. To do so, the researchers laboriously select items that fit specified criteria (i.e., differing on an explanatory variable of interest, while being closely matched on a number of control variables)—presumably by starting from some rough-hewn item lists and iteratively improving their selections by adding and removing individual items to make the lists better matched in the control dimensions. The need to create matched subsets of items is widespread, though, and the manual process suffers from a number of problems: manual selection is tedious and painstaking work (Cutler, 1981), precludes even the logical possibility of reporting every aspect of the reasoning in selection, may not result in well-matched stimuli (Van Casteren & Davis, 2007), can be prone to error (Armstrong, Watson, & Plaut, 2012), and may introduce bias (Forster, 2000).

As such, several algorithmic approaches to generating matched subsets have been proposed recently, including MATCH (Van Casteren & Davis, 2007), SOS (Armstrong et al., 2012), and BALI (Coupé, 2011). The underlying computational problem is provably difficult, and these algorithmic approaches vary in the ways—generally, which forms of randomization and heuristic approaches—that they use to handle that difficulty. MATCH finds a set of (yoked) pairs that are similar in control dimensions using backtracking and pruning; SOS (“stochastic optimization of stimuli”) finds sets of items that are close in aggregate by starting from a random seed and making randomized local improving swaps using simulated annealing. There are also approaches based on genetic algorithms (BALI, “balancing lists”), as well as an algorithmic tool based on *k*-means clustering to give computational support for manual selection of items (Guasch, Haro, & Boada, 2017).

An alternate approach—also founded on the idea of selecting a carefully chosen subset of a large population, although here in a *post hoc* way—is based on the statistical technique of *matching* in observational studies (Austin, 2011; D’Orazio, Zio, & Scanu, 2006; Gu & Rosenbaum, 1993; Rosenbaum & Rubin, 1983). In this scenario, the goal is typically to infer the effect of an intervention in a population in settings where the assignment of individuals to the treatment group is chosen by some external decision-maker rather than being specified by the researcher; thus the allocation may be biased in any number of ways. As such, matching uses *post-*intervention subset selection to simulate a randomized controlled trial: a set of untreated controls is chosen from a large population of candidate untreated individuals, so that the selected subset matches the set of treated individuals with respect to the covariates. There are multiple approaches to selecting the matched control set, but *propensity score matching* (*PSM*), which aims to match the “propensity”—the probability of treatment conditioned on covariate values—is perhaps the most prominent. This approach applies, and is widely used, under reasonable assumptions about the way that individuals’ treatment decisions were made and about the characteristics of the broader population, including the supposition that the population contains individuals who vary sufficiently on the measures of interest. (See Hirano, Imbens, and Ridder (2003) and Caliendo and Kopeinig (2008) for more on the assumptions and implementation of PSM.) Indeed, an algorithmic tool for *ex ante* item selection based on PSM has recently been proposed (Huber, Dietrich, Nagengast, & Moeller, 2017).

### The “Divide, Select, and Test” methodology

In both the experimental and observational methodologies just outlined, the researcher is seeking to assess the impact of a categorical variable *x*, typically representing population 1-vs.-population 2 membership or a treatment/no-treatment decision, on an outcome *y*. But a balanced-subset methodology is also sometimes used in conjunction with a “high–low split” when trying to understand how a continuously measured *explanatory variable x* predicts a *response variable y*, again while controlling for *d* different *control variables c*_1_, *c*_2_, *…, c_d_*. A high–low split is also sometimes called *dichotomization*. (In the examples described in the previous section, the researcher or some other decision-maker *assigns* the value of *x* to carefully chosen members of the population, or there are two discrete groups with different *x* values; here, the value of *x* is a continuously varying quantity that differs across members of the population.) In dichotomization, the population is divided into two sets, *L* (low *x*) and *H* (high *x*), and the *y* values of *L* and *H* are compared.

When dichotomization is combined with balanced-subset selection, sets *A* ⊆ *L* and *B* ⊆ *H*—that is, a subset *A* of the low values *L*, and a subset *B* of the high values *H*—are selected so that two conditions hold:

- *|A|* = *|B|* (that is, the sizes of *A* and *B* are identical); and
- *A* and *B* match, on average, with respect to each of the control variables *c*_1_, *c*_2_, *…, c_d_*.

The values of *y* in *A* and *B* are then compared. This comparison is typically done using a *t*-test, which evaluates whether *A* and *B* differ significantly in their *y* values. We refer to this three-part methodology as “divide, select, and test” (DS&T): *divide* the population based on *x* into *L* and *H*; *select* sets *A* ⊆ *L* and *B* ⊆ *H* that match on *c*_1_, *c*_2_, *…, c_d_*; and *test* using a *t*-test whether *A* and *B* differ on *y*.

Under circumstances in which dichotomization is appropriate, existing algorithmic implementations (Armstrong et al., 2012; Coupé, 2011; Huber et al., 2017; Van Casteren & Davis, 2007) are well-suited to efficiently selecting subsets while avoiding bias. However, dichotomization has well-known limitations, including a cost in statistical power (Cohen, 1983; Gelman & Park, 2009; Iacobucci, Posavac, Kardes, Schneider, & Popovich, 2015a, 2015b; McClelland, Lynch Jr., Irwin, Spiller, & Fitzsimons, 2015; Rucker, McShane, & Preacher, 2015). Here, we seek to assess the reliability and robustness of the DS&T approach by comparing it to other methods by which stimuli could be selected and effects tested.

### “Divide, Select, and Test” in psycholinguistics

The DS&T methodology is used frequently in the psycholinguistics literature to support claims about how particular lexical properties affect the perception and production of spoken and written words. Specifically, in the context of spoken-word recognition (SWR) tasks, papers using DS&T have informed much of our understanding about lexical characteristics that make a word easier or harder for listeners to recognize. It has long been known that the greater the *frequency* with which a word appears in natural language, the more quickly and accurately it is recognized (Connine, Titone, & Wang, 1993; Luce & Pisoni, 1998; Savin, 1963). Other research has explored effects based on the *competitors* of the word—that is, other words that are within a single phonemic insertion, deletion, or substitution of the word itself. For example, the competitors of *car* include *Carl* (an insertion); *are* (a deletion); and *bar, core, care*, and *call* (substitutions). DS&T studies first explored the most basic measure of competition on a word’s recognizability, namely the word’s *total number of competitors*: the more competitors a word has, the harder it is to recognize, even controlling for the word’s frequency (Goldinger, Luce, & Pisoni, 1989; Luce & Pisoni, 1998).

More subtle metrics about a word’s competitors have also been investigated, including the *clustering coefficient*, the fraction of pairs of the word’s competitors that are themselves competitors of each other. For example, if *Carl, are, bar, core, care*, and *call* were the only six competitors of *car*, then *car* would have a clustering coefficient of 3/15 = 0.2: of the 15 pairs of words that are competitors of *car*, the only three pairs that are themselves competitors are *Carl/call* (a deletion/insertion), *are/bar* (another deletion/insertion), and *core/care* (a substitution). (We treat clustering coefficient as undefined for any word with fewer than two competitors.) DS&T has also been used to show that high clustering coefficient is negatively associated with spoken- (Altieri, Gruenenfelder, & Pisoni, 2010; Chan & Vitevitch, 2009) and written-word (Yates, 2013) recognition, even controlling for frequency and number of competitors.

In the present work, we concentrate on the effects of frequency, number of competitors, and clustering coefficient, but a large number of other lexical properties have been evaluated throughout the psycholinguistics literature. Other experiments using DS&T have concluded the presence of a significant effect from a host of other word properties, even controlling for previously known effects. These properties include the perceived subjective *familiarity* of the word (Connine, Mullennix, Shernoff, & Yelen, 1990); the *phonotactic probability* of the word [the frequency with which a particular segment occurs in a given position in a word (Vitevitch & Luce, 1998; Vitevitch, Luce, Pisoni, & Auer, 1999)]; the *average frequency of occurrence of a word’s competitors* (Luce & Pisoni, 1998); the number of *onset competitors* [competitors that result from a substitution of the word’s first phoneme (Vitevitch, 2002)]; the competitors’ *spread* [the number of phonemic positions in which a substitution creates another word (Vitevitch, 2007)]; its *2-hop density* [the density of connections among competitors and their competitors (Siew, 2016)]; and the *isolation point* of the word [how many phonemes into the word one has to go before the word is uniquely identified (Marslen-Wilson & Tyler, 1980)]. SWR provides a convenient venue for testing whether effects observed in small samples of items generalize to the larger population of items because data collection is sufficiently tedious that researchers tend to minimize the number of stimuli, but not so tedious that it is not possible to collect data on a much larger set of items.

## Materials

SWR data were collected using standard methods. Stimuli were recorded by a native English speaker with a standard Midwestern American accent in a double-walled sound-attenuating chamber and leveled to total RMS in Adobe Audition. Participants were native English speakers who reported normal hearing and vision, recruited from the Washington University in St. Louis subject pool. Informed consent was obtained from participants and the research was approved by the Institutional Review Board where the SWR data were collected. Subsets of a list of 1120 monosyllabic words were presented to 94 participants (such that each word was presented to 30–32 participants), who attempted to identify the words by typing them. Word order was randomized. Stimuli were presented through headphones using E-Prime in six-talker babble at signal-to-noise ratio of –5. Both homophones and unambiguous nonwords whose obvious phonology matched the correct orthography (e.g., “turse” for “terse”) but no other deviations in spelling (e.g., pluralizations) were scored as correct. Starting from the correct/incorrect tags from the original dataset, we manually flipped a small number of correct/incorrect designations based on the identical-pronunciation criterion, changing a total of 362 participant–stimulus pairs out of 33510, less than 1.1% of the data. (To ensure these changes did not systematically affect the outcomes, we also ran all of our analyses on the uncorrected data; the results were nearly identical to those reported here.) Of the 1120 words used as stimuli, 38 were not consonant-vowel-consonant words and 1 (“bass”) was pronounced differently than its form in the English Lexicon Project (ELP; Balota et al., 2007), the dataset we use to calculate lexical characteristics. Thus, the analyses reported here were conducted on the remaining 1081 words, referred to here as the SWR1081 dataset. The dataset and all code necessary to run our analyses is available online through the Open Science Framework at: https://osf.io/x73dy/.

**Figure 1:**
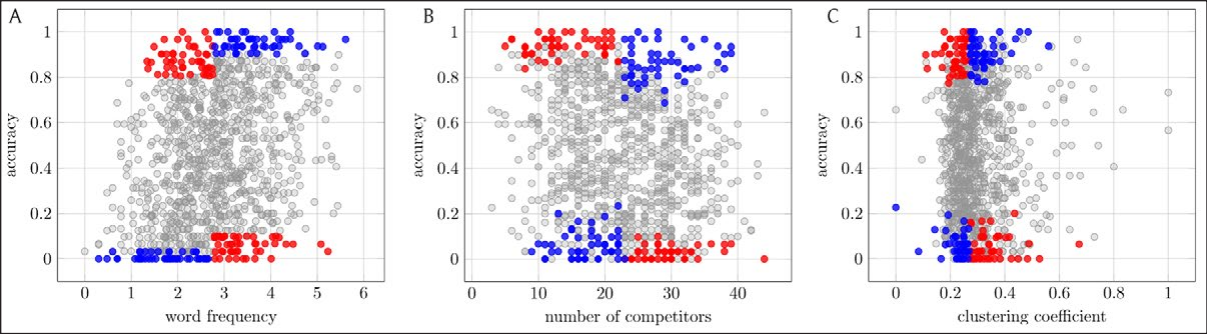
Maximizing the apparent positive and negative effect of an explanatory variable on SWR accuracy. Each panel shows all 1081 words in SWR1081, plotting for each word *w* an explanatory variable against the response variable *accuracy*, the fraction of participants who correctly identified this word when it was presented in a noisy environment. The explanatory variables are **(A)** the log frequency of *w* in a large corpus of natural text (Brysbaert & New, 2009), **(B)** the number of competitors of *w* in the ELP lexicon (Balota et al., 2007), and **(C)** the clustering coefficient of *w* in the ELP lexicon. (Every word in SWR1081 has at least three competitors, so clustering coefficient is well defined.) Each panel identifies two pairs of 50-word subsets {*A*_1_, *B*_1_} (blue; positive effect) and {*A*_2_, *B*_2_} (red; negative effect). Each pair of color-matched subsets controls for all explanatory variables in previous panels: in (B), *A_i_* and *B_i_*’s average frequency (in *z*-score) differ by less than *δ* = 0.05, and likewise in (C) for both average frequency and number of competitors. Among all such δ-balanced 50-element subsets, the displayed subsets show the largest possible difference (positive and negative) in *y* for low-*x* and high-*x* words. (See Supplementary Materials for how these subsets are computed.)

**Figure 2:**
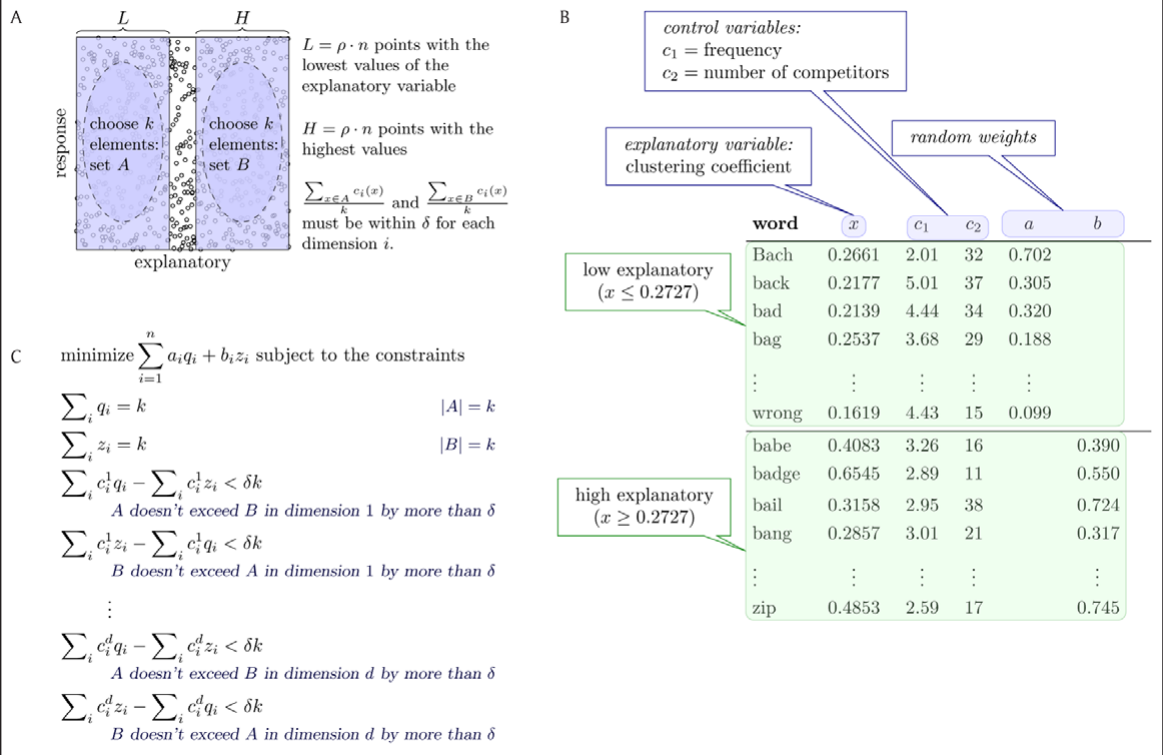
A schematic of the selection process, with parameters *k* (size of chosen subsets), ρ (fraction of data considered “high” or “low”), and δ (tolerance in control variables). **(A)** We must choose 2*k* of *n* given data points, in two equal-sized sets *A* and *B*, where *A* is chosen from among the ρ · *n* points with lowest explanatory variable values and *B* is chosen from among the ρ · *n* highest points. In every control dimension *c_i_*, the elements of *A* and *B* are, on average, within δ. **(B)** A particular example of this input data in a SWR context, with data from the ELP lexicon (Balota et al., 2007; Brysbaert & New, 2009). The weights *a_i_* and *b_i_* are chosen uniformly at random from [0,1]. The desired solution is the lightest-weight pair of sets *A* and *B* (with respect to these particular *a* and *b* weights) that satisfies the control-dimension constraints. **(C)** The integer linear program (ILP) used to compute the solution. We define variables *q_i_* ∈ {0, 1} and *z_i_* ∈ {0, 1} indicating whether to include a point in *A* and *B*, respectively. Solving the ILP finds optimal values of *q_i_* and *z_i_*. Fresh random weights are chosen in each run of the algorithm.

In all analyses, including the integer linear program (ILP) in Figure 2(c), we use the *z*-scores of each numerical field (e.g., word frequency, number of competitors, clustering coefficient, accuracy) with respect to the full SWR1081 dataset. This choice puts all variables on comparable scales. However, for ease of interpretation, Figure 1 and 2(b) show the raw numbers in the dataset.

## Analyses and Results

In the present work, we show that DS&T-based analysis can lead to conclusions that are not well supported by data, including both the possibility of false positives (observing an effect through DS&T that is not present in the full population) and false negatives (frequently missing an effect through DS&T that is present in the full population). We also show that using either linear regression or linear mixed effects models on a subset of items provides greater statistical power than DS&T in SWR1081; we show similar results for linear regression on several synthetic datasets. (Generating a synthetic dataset suitable for analysis by linear mixed effects models requires more assumptions than we were willing to make in our generative process. Specifically, linear mixed effects models require participant-by-item data. Generating such synthetic data relies on a large number of parameters and assumptions about both participants and items, including the shape of distributions; many choices of parameters and assumptions would be consistent with the limited data that we would try to match.)

### Demonstrating both positive and negative effects of the same variable

As just described, DS&T-based analysis has been used to argue that certain properties of words in the lexicon (with each of these properties serving as a candidate explanatory variable *x*) can predict human performance in recognizing those words (with recognition performance serving as the response variable *y*). Using the DS&T methodology with prior knowledge of the response variable, however, one can show contradictory results about the influence of an explanatory variable. Typically, of course, in a SWR experiment, the researcher would choose the sets *A* and *B*—the selected subpopulations of *L* and *H*, the low *x* and the high *x* segments of the population—*before* experimentally gathering the *y* values. One would want to choose these sets in advance because collecting data with human participants and their responses to lexical stimuli is resource intensive. And one would also want to select *A* and *B* in advance to ensure the moral equivalent of a double-blind study: it would be possible to put one’s “thumb on the scale” if the *y* values of *L* ∪ *H* were known at the time of selection of *A* and *B*. [Having collected *y* values first would make the methodology much more similar to the observational setting of matching; for similar reasons, the matching literature counsels refraining from looking at outcome values when doing selection to avoid the risk of implicit bias in selecting which individuals to include in the dataset (King & Nielsen, 2016; Rubin, 2008).]

To illustrate this possibility of contradictory results, we use the SWR1081 dataset to ask how the selection of words affects the results that we observe. Following precisely the divide-and-select methodology (though peeking at the values of *y*), we are able to build two different pairs of balanced subsets {*A*_1_, *B*_1_} and {*A*_2_, *B*_2_}, where {*A*_1_, *B*_1_} demonstrates a strong positive effect of *x* on *y* and {*A*_2_, *B*_2_} demonstrates a strong negative effect of *x* on *y*: that is, (i) the sets *A*_1_ and *B*_1_ are matched in all control dimensions (to within a specified tolerance, which we denote by δ) and *y*(*A*_1_) is much less than *y*(*B*_1_), whereas (ii) the sets *A*_2_ and *B*_2_ are also matched in all control dimensions but *y*(*A*_2_) is much greater than *y*(*B*_2_) (Figure 1).

### Testing the effects of explanatory lexical variables using many different pairs of balanced subsets

We just showed that it is possible to find two pairs of extreme balanced subsets of SWR1081 showing highly positive or negative effects of an explanatory variable on a response variable. We now turn to generating *many* different pairs of balanced subsets through algorithmic means. This problem is a concrete, algorithmic task:

- We are given two populations *L* and *H*^1^. For each *x* ∈ *L* ∪ *H*, we are given control-variable values *c*_1_(*x*), *…, c_d_*(*x*). We are also given a target set size *k*, and a tolerance δ > 0.
- We must find sets *A* ⊆ *L* and *B* ⊆ *H*, subject to two constraints:
  - *|A|* = *|B|* = *k* (that is, both *A* and *B* have size equal to *k*); and
  - for each control dimension *i*, the difference in *A* and *B*’s average values in that control dimension is within δ:

\left| {\frac{{\sum\nolimits_{x \in A} {{c_i}(x)} }}{{|A|}} - \frac{{\sum\nolimits_{x \in B} {{c_i}(x)} }}{{|B|}}} \right| \le \delta .

When there are a large number of control dimensions, selecting *A* ⊆ *L* and *B* ⊆ *H* is a tedious and difficult task (even if, unlike in the previous section, we do not try to push the response variable in either direction); thus we seek a general, systematic, and unbiased procedure to choose *A* and *B*.

This problem is intractable in general—it is NP-hard to solve (Garey & Johnson, 1979; Kleinberg & Tardos, 2005) (see Supplementary Materials)—but for practical instances of reasonable size, this problem can be solved using an Integer Linear Program (ILP) and an off-the-shelf ILP solver called Gurobi (Gurobi Optimization, Inc., 2015; Papadimitriou & Steiglitz, 1982). The problem that we solve with our ILP is similar to the one solved in selection via the algorithmic approaches to subset selection detailed above, but we have chosen to design an algorithm to solve precisely the problem that corresponds to the DS&T methodology appearing regularly in the SWR literature that (i) optimally solves the item-selection problem and (ii) naturally allows the calculation of many pairs of balanced sets *A* and *B*.^2^ We can achieve (ii)—that is, we can produce many different solutions for the same instance of the problem—by assigning randomly chosen weights to each element of *L* ∪ *H*, and defining an ILP that selects, from among all sets satisfying the balance conditions, those sets *A* and *B* whose total weights are minimized (Figure 2). In this way, we are able to rapidly construct many different pairs of balanced sets *A* and *B*.

Papers on word recognition have used DS&T to claim effects on human recognition performance, using a single balanced pair of low/high word sets in each experiment: word frequency [high frequency corresponds to high recognizability (Connine et al., 1993; Luce & Pisoni, 1998; Savin, 1963)], number of competitors [many competitors corresponds to low recognizability (Goldinger et al., 1989; Luce & Pisoni, 1998)], and clustering coefficient [high clustering corresponds to low recognizability (Altieri et al., 2010; Chan & Vitevitch, 2009; Yates, 2013)]. However, by sampling over many different balanced pairs of word sets, we see that the apparent strength of the effect on recognition accuracy varies dramatically across the chosen subsets.

**Figure 3:**
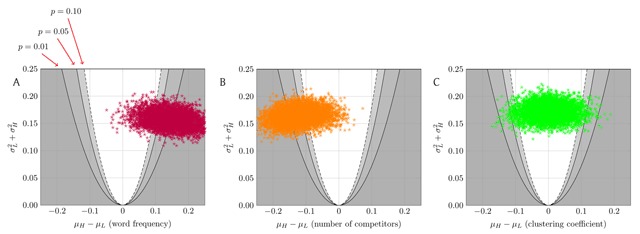
The result of 5000 runs of our ILP, with *k =* 50 words per subset, δ = 0.05 tolerance for control variables, and ρ = 0.5 (dichotomizing on the median). Each point in each panel corresponds to a single run of the ILP to select sets *A* and *B*; the point plots the difference in mean recognition accuracy between *A* and *B*, vs. the sum of the variances of the recognition accuracies in *A* and *B*. The parabolas correspond to significance levels in a *t*-test on *A* vs. *B*. **(A)** The effect of frequency on recognition; 72.6% of these runs show that higher frequency is associated (*p* < 0.05) with more accurate recognition. **(B)** The effect of number of competitors; 53.8% of these runs show that having more competitors is associated (*p* < 0.05) with less accurate recognition. **(C)** The effect of clustering coefficient; 2.1% of these runs show an effect of clustering coefficient on recognition (*p* < 0.05), split between showing positive and negative effects. All experiments were controlled as in Figure 1. (See Figure S1 for the variant of this analysis that tests the effect of each variable while controlling for the other two.)

Specifically, we ran 5000 DS&T experiments using the ILP in Figure 2 for the division/selection steps for these three lexical properties (Figure 3). The positive effect of high word frequency and the negative effect of a large number of competitors are largely evident and support prior research. (Frequency: 3632 of 5000 runs significant at *p* < 0.05; competitors: 2690 of 5000.)

However, in contrast to the results claimed in the literature, only in rare runs of the ILP does clustering coefficient show a significant effect on recognition accuracy when controlling for word frequency and number of competitors (Figure 3, 105 of 5000 runs significant at *p* < 0.05). Furthermore, even those rare runs that show a clustering-coefficient effect are split on the direction of effect (41 of the 105 show a negative effect; 64 of 105 show a positive effect).

### Comparing DS&T, linear regression, and linear mixed effects models

The literature includes at least two alternative approaches to the DS&T methodology. Researchers may instead opt to run correlations or linear regressions on continuously valued datasets (Luce & Pisoni, 1998; Strand & Sommers, 2011). Using linear regressions differs from DS&T in both how stimuli are selected—by including words that vary continuously on the explanatory variable rather than binning high and low sets—and how the analysis is conducted—by statistically, rather than experimentally, controlling for the influence of control variables.

Another approach is to use linear mixed effects models (LMEMs), which provide a general, flexible approach to dealing with nested or hierarchical data (e.g., the fact that each word is identified by multiple participants). In these analyses, the LMEMs take raw, trial-level word-recognition correct/incorrect tags as input, rather than averaged accuracy values that collapse across participants. Like regressions, LMEMs can accommodate continuously valued data and statistically control for the influence of other variables. Much has been written about the benefits of the LMEM approach (Baayen, Davidson, & Bates, 2008; Clark, 1973; Jaeger, 2008); most notably for our project, an advantage of LMEMs over linear regressions is that LMEMs have access to information about variability at both the item and participant level, which may help reduce the error variance that can arise in large-scale studies (Sibley, Kello, & Seidenberg, 2009).

To assess these different approaches, we compared the DS&T methodology (i.e., using the ILP with random weights to select *A* and *B* with sizes *|A|* = *|B|* = *k* and using a *t*-test to look for a difference between *A* and *B*), denoted by **ILP‖*t*-test**, against four other methodologies. First, we considered two other ways of analyzing the sets produced by the ILP:

**ILP‖lin-reg:** ILP selection as just described, but using linear regression on the 2*k* elements of *A* ∪ *B* to test for an effect. (This methodology is appropriate only when ρ = 0.5, because an unpopulated central range of *x* values would violate the assumptions of linear regression.)

**ILP‖LMEM:** ILP selection as above, but using LMEM on the 2*k* elements of *A* ∪ *B* to test for an effect. In these models, participants and items were entered as random effects and the explanatory and control variables were entered as fixed effects. Given the large number of simulations that we ran, and the well-known problems with convergence for models that include them, by-participant random slopes for the lexical variables were not included. Omitting by-participant random slopes can increase the rate of false positives, but, as we shall see, our most interesting results are about the *low* positive rate associated with clustering coefficient. We used a logit linking function given that the explanatory variable (word-recognition accuracy) was binomial.

**Figure 4:**
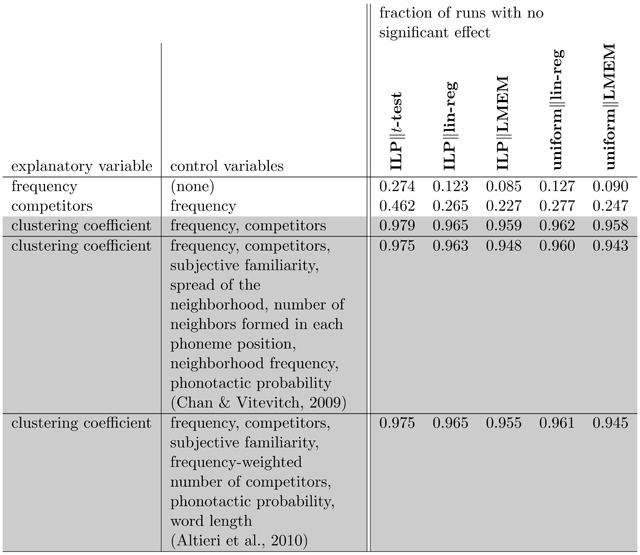
Comparison of power of various testing methodologies. We considered two explanatory variables with significant effect on recognition accuracy in SWR1081, as determined by linear regression and LMEM applied to the entire dataset (*p* < 0.001 for both frequency and competitors). We measured how often five different testing methodologies failed to detect the correct effect (at the *p* = 0.05 level) in subpopulations of 100 total words, over 5000 trials. ILP selection follows Figure 2; uniform selection chooses the same number of elements by uniform random sampling. We tested for a relationship using either a *t*-test comparing the low and high sets’ response-variable values, via linear regression (in either case controlling for the listed control variables), or LMEM. The first two rows show settings in which there is a true effect (measured on the whole dataset); here, linear regression and LMEM correctly detect an effect more frequently than *t*-tests. When used with linear regression or LMEM, ILP performed slightly better than uniform sampling. For contrast, the last three rows show a setting in which there is no apparent relationship on the whole dataset (*p* > 0.5 using both linear regression and LMEM), where all three methodologies showed no effect >94% of the time. The last two of these rows perform the clustering coefficient analysis while controlling for a much larger list of variables, following the methodology of Chan and Vitevitch (2009) and Altieri et al. (2010). Note that we did not directly attempt to correct for multicollinearity among variables; however, given the close similarity of the analyses in the last three rows of the table, which correspond to very different settings of control variables, and the fact that all of the ILP analyses (which control for covariates via selection rather than only statistically) are consistent with the uniform analyses, multicollinearity is not likely to substantially affect the results.

We tested each method’s prediction of the effect of word frequency and number of competitors (Figure 4). We conducted the *t*-tests under the assumption of equal variance to make them more analogous with linear regression. In these experiments, **ILP‖*t*-test** had a higher likelihood (≈2 to 3 times) of failing to detect a significant effect than **ILP‖lin-reg** and a higher likelihood (again, ≈2 to 3 times) of failing to detect a significant effect than **ILP‖LMEM**. While the dichotomization literature has long recognized the gain in power of linear regression over *t*-tests for complete datasets (Cohen, 1983; Gelman & Park, 2009; Iacobucci et al., 2015a, 2015b; McClelland et al., 2015; Rucker et al., 2015), here we are testing effects on carefully selected subpopulations; the results in Figure 4 suggest that a *t*-test remains far more likely than linear regression or LMEM to miss a true effect, even for intentionally chosen balanced subsets.

We then compared the use of linear regression and LMEM on sets selected via ILP to sets selected in a different way: via pure randomization—i.e., choosing the same number of elements uniformly at random from the full population. (The *t*-test does not apply for the uniform selection mechanism, as it does not select two distinct populations.) Uniform selection yields two further possible analyses:

**uniform‖lin-reg:** we use uniform random sampling to select 2*k* elements from the entire population, and then use linear regression as above to test for an effect.

**uniform‖LMEM:** we use uniform random sampling to select 2*k* elements from the entire population, and then use LMEM as above to test for an effect.

Comparing **uniform‖lin-reg** and **uniform‖LMEM** to their ILP-generated counterparts, we see only a small difference in power: the ILP selection strategy has a mild advantage in the rate of runs with significant effects (for both frequency and number of competitors, both **ILP‖lin-reg** and **ILP‖LMEM** outperform their uniform counterparts). (See Figure 4 and also Figure S1.)

Using both linear regression and LMEM on the entire SWR1081 dataset suggests that, in keeping with Figure 3, word frequency and number of competitors do matter in word recognition (*p* < 0.001 for both variables and both analyses). We also tested the effect of clustering coefficient on recognizability, which does not have a significant effect in the full population (*p* > 0.5 for both linear regression and LMEM). When controlling for frequency and number of competitors, all five of our analysis methods do not find a relationship between clustering coefficient and recognition accuracy in the vast majority of runs (for each analysis method, fewer than 6% of runs found a relationship at the *p* = 0.05 level). To be consistent with previous published work that has shown significant effects of clustering coefficient on SWR (Altieri et al., 2010; Chan & Vitevitch, 2009) we also ran the same analyses with a larger set of control variables to match the conditions of the previous studies (note that these studies used lexicons other than ELP, so our values for number of competitors and clustering coefficient are close but not numerically identical to theirs); again, over 94% of runs fail to identify a significant relationship between clustering coefficient and accuracy.

**ILP‖*t*-test** was less likely than the other approaches to detect significant effects present in the full population (frequency and number of competitors). But the lower power of **ILP‖*t*-test** cannot be fully attributed to it being a conservative approach; when testing the effect on accuracy of clustering coefficient—which has no apparent explanatory effect on the response variable—the ILP-based selection of balanced sets still yields false positives >2% of the time, suggesting that such sets can be generated accidentally.

### DS&T versus uniform selection on synthetic data

We have considered several methodologies for testing the effect of number of competitors (*x*) on word-recognition accuracy (*y*) while controlling for word frequency (*c*) in the SWR1081 dataset. To ensure that the issues with the DS&T methodology were not specific to the particular psycholinguistic dataset that we considered, we also attempted to replicate our results in a number of synthetic datasets. These synthetic datasets were designed to match the SWR1081 dataset in size and in relationships among these three variables—but with data that are drawn precisely from a multivariate normal distribution.

**Figure 5:**
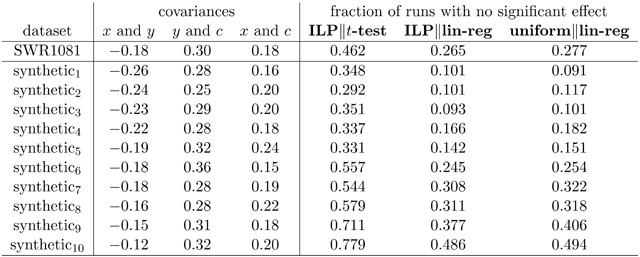
Analysis of SWR1081 and ten synthetic datasets generated to have (approximately) the same covariance matrix. The ten synthetic datasets are sorted in decreasing order of the strength of the relationship between *x* and *y*.

Specifically, using the covariances from the SWR1081 dataset (shown in the first row of Figure 5), we generated 10 synthetic datasets as follows: we generate *n* = 1081 data points, where each generated point is drawn from a multivariate normal distribution with all means equal to *0* and whose covariance matrix matches that of the *z*-scores of SWR1081. (Note that the covariance of the *generated* synthetic datasets does not match SWR1081 precisely, because the synthetic datasets by definition are randomly constructed and do not precisely achieve their expected values.) We then ran the same *t*-test and regression analyses as in Figure 4 on all ten synthetic lexicons. Given the similarity of the results generated by linear regression and LMEM and the hard-to-justify assumptions necessary for generating synthetic trial-level data that accurately represents both item- and participant-level variability, we limited the analysis of the synthetic data to *t*-tests and linear regression.

The results are shown in Figure 5. We see the same broad patterns in the synthetic datasets as we do in SWR1081. First, **ILP‖*t*-test** has a higher likelihood of failing to detect a significant effect than **ILP‖lin-reg** or **uniform‖lin-reg**—generally by a factor of ≈2–3. Second, **ILP‖lin-reg** and **uniform‖lin-reg** have broadly similar false-negative rates. (There again appears to be a slight benefit for **ILP‖lin-reg** over **uniform‖lin-reg**, but the difference is modest.)

While the relative differences among the testing methodologies are fairly consistent across datasets, the raw values of the probability of detection of an effect varies across the ten synthetic datasets. The difference tracks the magnitude of the relationship between *x* and *y*; unsurprisingly, weaker correlations in the full population are more likely to be missed in the selected subsets.

## Discussion

Taken together, our results show that DS&T increases the likelihood of false negatives without reducing the false-positive rate. DS&T also notably lacks transparency in how word sets are generated and matched: the key step of selecting which words from the lexicon to study is sublimated, and the principles by which selections were made are typically left opaque when the research is published. These choices have the potential to qualitatively affect the conclusions of a study, and thus serve as another researcher degree of freedom. Similar concerns have been raised about studies using matching, particularly PSM (Arceneaux, Gerber, & Green, 2010; King & Nielsen, 2016; LaLonde, 1986). Unlike in matching-based studies about the effect of an intervention, though, we have a different option available: simply leave the continuous variable continuous. At a time when others are calling for larger participant sample sizes (Anderson & Maxwell, 2017), we also recommend using larger sets of stimuli that more completely represent the population of stimuli—and analyzing the results using continuous statistics.

Continuous statistical methods—using linear regression or LMEMs on a randomly selected subset of stimuli—provides greater power and transparency of process than DS&T. Of course, these methods may come with their own challenges including how to deal with multicollinearity among predictor variables (Farrar & Glauber, 1967), how to make decisions about which potential covariates to include, and, in the case of LMEM, how to specify a random effects structure (Barr, Levy, Scheepers, & Tily, 2013). There may also be circumstances in which continuous statistics are not available or relevant: in many medical and policy-based studies, groups are truly categorical (e.g., control and experimental). In such cases, existing algorithms (Armstrong et al., 2012; Coupé, 2011; Huber et al., 2017; Van Casteren & Davis, 2007) can be viewed as an alternative to dynamic allocation or matching techniques to assign individuals to treatment groups. Even here, though, care must be taken with statistical tests deployed in studies using dynamic allocation (Pond, 2011).

There appears to be a slight benefit in the true-positive rate of **ILP‖lin-reg** over **uniform‖lin-reg**: the ILP-based methodology modestly outperforms the uniform approach in SWR1081 and in 9 of the 10 synthetic datasets. **ILP‖LMEM** also outperforms **uniform‖LMEM** in SWR1081. This modest improvement may derive from the fact that the ILP necessarily selects a subpopulation that has a good spread of *x* values, whereas a uniform sample may have only a narrow swath of elements. [Note that ensuring this kind of variation is a feature that the SOS algorithm can support directly, by preferring subsets that have higher entropy in a given variable (Armstrong et al., 2012).]

Despite the intuitive nature of DS&T—all three of its components (dichotomization, controlling for covariates using subset selection, and *t*-tests) are seemingly innocuous—their combination not only weakens statistical power but also fails to eliminate the risk of false positives. Concretely, the previously published conclusions about the effect of clustering coefficient on spoken-word recognition are not supported by our analysis; we do not see evidence that clustering coefficient plays any significant role in recognition accuracy. Given the current replication crisis in psychology (Loken & Gelman, 2017; Open Science Collaboration, 2015), these results indicate that a certain attractive statistical approach in fact can lead to erroneous conclusions or suggest an unwarranted degree of confidence. DS&T approaches remain common, but compelling alternatives are appearing in the form of large-scale mega-studies on both written- (Balota et al., 2007) and spoken-word (Tucker et al., 2018) recognition. The flexibility afforded by DS&T in choosing which data points to study allows a researcher to analyze a subpopulation that may be atypical; conclusions about that subpopulation do not validly imply anything about the population as a whole.

## Additional File

The additional file for this article can be found as follows:

10.5334/joc.51.s1 Supplementary Materials. Further details on the data and the analysis.

## Footnotes

In this paper, for a positive parameter ρ *≤* 0.5, we construct *L* as the ρ-fraction of the population with the lowest values of *x*; similarly, *H* is the ρ-fraction of the population highest in *x*. Throughout this paper, we use ρ = 0.5, a median split. Note that using ρ = 0.5 means it is conceivable that words that have precisely the median value on the explanatory value could appear in both the *L* and *H* sets, although for SWR1081 and for the explanatory variables considered in this paper, this situation did not happen to occur.

The fact that we optimally solve the item-selection problem (despite the problem’s intractability) means that our ILP-based algorithm reliably produces the same output in every instantiation, though it comes at the cost of additional computation time compared to existing algorithmic approaches like SOS and MATCH (Armstrong et al., 2012; Van Casteren & Davis, 2007), which use randomization and heuristic optimization techniques. Note too that these existing tools also support a wider range of constraints and quality measures on the selected sets. For example, matching-based approaches—like MATCH or PSM—generally aim for individual-level matches for each treated individual, not just “on average” matches between the treated and untreated populations; our algorithm only seeks group-level similarity.
