# Supplementary material for: The Danger of Testing by Selecting Controlled Subsets, with Applications to Spoken-Word Recognition
Source: J Cogn. 2019 Jan 24;2(1):2. doi: 10.5334/joc.51 (PMC6634384; doi:10.5334/joc.51)
Supplement: Supplementary Materials. — Further details on the data and the analysis. [file joc-2-1-51-s1.pdf]

Supplementary Materials

Descriptive Data

	mean	standard deviation	range
accuracy	0.45	0.29	0–1
frequency	2.78	1.01	0–5.86
number of competitors	22.52	7.89	3–44
clustering coefficient	0.29	0.1	0–1

Integer Linear Programs to choose subsets of words

In the main text, we describe an algorithm to identify two sets A and B that are different with respect to an explanatory variable x (A comes from the part of the population with "low" x, and B from the "high" x subset) and such that A and B are balanced with respect to a given list of control variables. In Figure 2, we describe how we compute the balanced subsets A and B, using an integer linear program (ILP). For an introduction to integer linear programming, see Papadimitriou and Steiglitz (1982). In Figure 2, we use the following ILP, for randomly chosen weights a and b:

$$\text{minimize} \sum_{i=1}^{n} a_i q_i + b_i z_i \text{ subject to the constraints that}$$

$$\sum_i q_i = k \qquad |A| = k$$

$$\sum_i z_i = k \qquad |B| = k$$

$$\sum_i c_i^1 q_i - \sum_i c_i^1 z_i < \delta k \qquad \textit{A doesn't exceed B in dimension 1 by more than } \delta$$

$$\sum_i c_i^1 z_i - \sum_i c_i^1 q_i < \delta k \qquad \textit{B doesn't exceed A in dimension 1 by more than } \delta$$

$$\vdots$$

$$\sum_i c_i^d q_i - \sum_i c_i^d z_i < \delta k \qquad \textit{A doesn't exceed B in dimension d by more than } \delta$$

$$\sum_i c_i^d z_i - \sum_i c_i^d q_i < \delta k \qquad \textit{B doesn't exceed A in dimension d by more than } \delta$$

Here the idea is that, after generating random weights a_i for each "low" word and b_i for each "high" word, we select the balanced sets of low and high words that are lightest with respect to the chosen weights. The weights a and b describe the "cost" of selecting particular words; by randomly choosing those weights differently from run to run of our ILP algorithm, different words have high cost in different runs of the algorithm, so the balanced pairs of subsets we compute consequently differ across the algorithm's runs.

In Figure 1, we carefully choose balanced sets to maximize the apparent effect of the explanatory variable by using the response variable as the guide to choosing sets, instead of randomly chosen weights: we seek the balanced sets of low and high words that are *most different with respect to the response variable.* To do so, we use a very similar ILP, but with a different objective function:

$$\text{maximize} \sum_{i=1}^{n} r_i q_i - r_i' z_i$$

where

$$r_i = \begin{cases} x(\text{word } i) & \text{if word } i \text{ is in the low set} \\ 0 & \text{otherwise} \end{cases} \qquad r_i' = \begin{cases} x(\text{word } i) & \text{if word } i \text{ is in the high set} \\ 0 & \text{otherwise,} \end{cases}$$

where $x(\text{word } i)$ denotes the response-variable value for word i. The remainder of the calculation is exactly as described in Figure 2.

Computational complexity of finding balanced subsets

We claim that finding balanced sets A and B is an intractable problem, in general. Here is a precise statement and outline of a proof, using a reduction from SubsetSum, a standard NP-complete problem (Garey & Johnson, 1979; Kleinberg & Tardos, 2005). Thus we would not expect to identify an efficient algorithm to solve the balanced subset problem; hence, the Integer Linear Program is an appropriate approach to solving the problem.

The specific algorithmic problem that we wish to solve (as described in the main text) is the following:

Definition 1. *The* BalancedSubset *problem is defined as follows.*

> **Input:** *two sets $A \subseteq \mathbb{R}^d$ and $B \subseteq \mathbb{R}^d$ of d-dimensional vectors, a positive integer $k \in \mathbb{Z}$, and a tolerance $\delta \geq 0$.*
>
> **Output:** *do there exist subsets $A' \subseteq A$ and $B' \subseteq B$, with $|A| = |B| = k$, such that, for every dimension $i \in \{1, 2, \ldots, d\}$,*

$$\left| \frac{\sum_{x \in A'} c_i(x)}{k} - \frac{\sum_{x \in B'} c_i(x)}{k} \right| \leq \delta?$$

To demonstrate the hardness of the general BalancedSubset problem, we will prove the hardness of a special case of it. Specifically, we consider the EqualHalves problem, which is the special case of BalancedSubset in which:

- $d = 1$: there is only one control dimension.
- $\delta = 0$: the tolerance is zero (so we have to find subsets that match exactly in that one dimension).
- $c_1(x) > 0$ for all x: the values of all points in that one control dimension are strictly positive.
- $|A| = |B| = 2k$: the given sets are identical in cardinality, precisely twice that of the desired subsets.

Here is the formal definition of EqualHalves:

Definition 2. *The* EqualHalves *problem is defined as follows.*

> **Input:** *two sets of positive integers A, B with $|A| = |B| = n = 2k$. (We permit duplicates in A and B.)*
>
> **Output:** *do there exist subsets $A' \subset A$ and $B' \subset B$, both with size k and with equal sums?*

We will prove the hardness of EqualHalves via reduction from SubsetSum; from this fact, we conclude the hardness of its generalization BalancedSubset.

Theorem 3. *The* EqualHalves *problem is* NP*-Complete.*

Proof. Via reduction from SubsetSum. An instance of the SubsetSum problem consists of a set of positive integers $X = \{x_1, x_2, \ldots, x_m\}$ and a target sum W. The goal is to determine whether there is an $X' \subseteq X$ whose sum is W. Without loss of generality, we can assume that $\sum_{x \in X} > W$. SubsetSum is well known to be an NP-complete problem (Garey & Johnson, 1979; Kleinberg & Tardos, 2005). (We permit duplicates in X, which doesn't affect the hardness of the problem.)

Given such an instance $\langle X, W \rangle$ of SubsetSum, construct an EqualHalves instance as follows. Define A to be the union of X and $m - 2$ zeroes. Define B to contain W and $2m - 3$ zeroes. We claim that $\langle X, W \rangle$ is a yes-instance of SubsetSum if and only if $\langle A, B \rangle$ is a yes-instance of EqualHalves.

($\Longrightarrow$) Suppose A' and B' is a solution to $\langle A, B \rangle$. The set A' must have a positive sum because strictly fewer than half of the elements of A are zero, and thus B' must contain W. Therefore the sum of elements in A' is W. Removing any zeroes from A' yields a subset of X whose sum is W.

($\Longleftarrow$) Suppose X' is a solution to $\langle X, W \rangle$. Generate A' by adding zeroes to X' until $|A'| = m - 1$. Let B' be W plus $m - 2$ zeroes. Both sets have size $m - 1$ and sum W. □

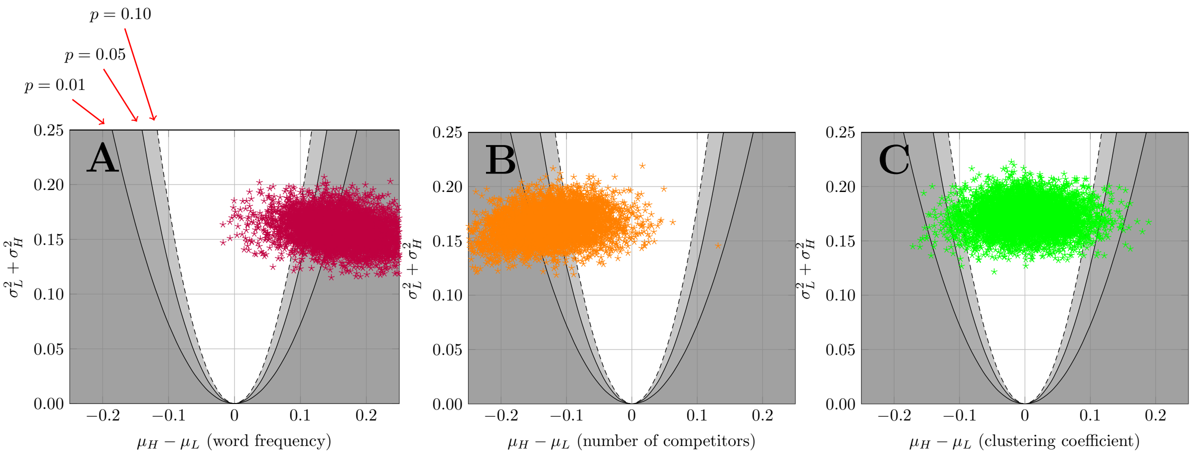

D

		fraction of runs with no significant effect				
explanatory variable	control variables	**ILP‖*t*-test**	**ILP‖lin-reg**	**ILP‖LMEM**	**uniform‖lin-reg**	**uniform‖LMEM**
frequency	competitiors, clustering coefficient	0.168	0.048	0.030	0.058	0.034
competitors	frequency, clustering coefficient	0.459	0.276	0.234	0.318	0.281
clustering coefficient	frequency, competitors	0.979	0.965	0.959	0.962	0.958

Figure S1: The result of 5000 runs of our ILP, with $k = 50$ words per subset, $\delta = 0.05$ tolerance for control variables, and $\rho = 0.5$ (dichotomizing on the median). All points shown in panels **(A)**, **(B)**, and **(C)** are as in Figure 3 from the main text, except that *here each panel controls for the explanatory variables shown in the other two panels.* [In the main text, we analyze the effect of (A) word frequency on accuracy; (B) the number of competitors on accuracy (controlling for frequency); and (C) clustering coefficient on accuracy (controlling for frequency and number of competitors). Here, we analyze the effect of (A) word frequency on accuracy (controlling for number of competitors and clustering coefficient); (B) the number of competitors on accuracy (controlling for frequency and clustering coefficient); and (C) clustering coefficient on accuracy (controlling for frequency and number of competitors). The results are qualitatively identical to the results in the main text.] Panels (A), (B), and (C) are the analogue of Figure 3, and Panel **(D)** is the analogue of Figure 4.
